# Fluorescence Mean‐Lifetimes of a Series of Small and Bright Fluorescent Dyes

**DOI:** 10.1002/bio.70392

**Published:** 2026-01-08

**Authors:** Leonardo De Boni, Klester dos Santos Souza, Melissa Machado Rodrigues, Bruna Nitzke Minuzzi, Marcelo Barbalho Pereira, Milton Katsumi Sasaki, Diogo Seibert Lüdtke, Tarso B. Ledur Kist

**Affiliations:** ^1^ Instituto de Física de São Carlos Universidade de São Paulo São Carlos SP Brazil; ^2^ Instituto de Química Universidade Federal do Rio Grande do Sul Porto Alegre RS Brazil; ^3^ Ciências Exatas e Engenharias Universidade de Caxias do Sul Caxias do Sul RS Brazil; ^4^ Instituto de Ciências Básicas da Saúde, Departamento de Bioquímica Universidade Federal do Rio Grande do Sul Porto Alegre RS Brazil; ^5^ Instituto de Física Universidade Federal do Rio Grande do Sul Porto Alegre RS Brazil; ^6^ LSO/PEA, Escola Politécnica Universidade de São Paulo São Paulo SP Brazil

## Abstract

Fluorescence mean‐lifetime is a photophysical parameter that is used in basic research and in many fields of applications. However, the parameter is not available in the literature for some small and bright fluorescent dyes, or at least it is difficult to find. Therefore, in the present work, the fluorescence mean‐lifetime of some small and bright fluorescent dyes was measured in aqueous buffered solutions. A few more dyes with an already known fluorescence mean‐lifetime are also included for comparison. The total list of five classes (scaffolds) and their respective dyes analyzed are as follows: (1) four rhodamines—5(6)‐carboxy‐X‐rhodamine (ROX), Rho 6G 3^+^ (N‐[2‐(2‐aminoethylamino)ethyl]rhodime 6G‐amide), Rho 6G 2^+^ (N‐(8‐amino‐3,6‐dioxaoctyl)rhodamine 6G‐amide), and TAMRA‐SE‐Gly (a carboxytetramethylrhodamine); (2) four fluoresceins—5‐aminofluorescein, 5(6)‐carboxyfluorescein (FAM), 6‐TET‐SE‐Gly (a tetrachlorocarboxyfluorescein), and 6‐HEX‐SE‐Gly (a hexachlorocarboxyfluorescein); (3) three pyrenes—8‐aminopyrene‐1,3,6‐trisulfonic acid, 8‐hydroxypyrene‐1,3,6‐trisulfonic acid, and pyrene‐1,3,6,8‐tetrasulfonic acid; (4) one borondipyrromethene—BODIPY FL (4,4‐Difluoro‐5,7‐dimethyl‐4‐bora‐3a,4a‐diaza‐s‐indacene‐3‐propanoic acid); and (5) one acridine—3,6‐dimethylaminoacridine (acridine orange). Fluorescence mean‐lifetimes were measured in one or more of the following mediums: 100 mM acetic acid/sodium acetate pH 4, water pH ~ 7, and 20 mM potassium tetraborate pH 10.

## Graphical abstract

Fluorescence mean‐lifetimes of a series of small and bright fluorescent dyes were measured in various solutions. It includes four rhodamines (Rho 6G 2^+^, Rho 6G 3^+^, ROX, TAMRA), four fluoresceins (5‐AF, FAM, 6‐TET, and 6‐HEX), three pyrenes, one borondipyrromethene (BODIPY FL), and one acridine (acridine orange).

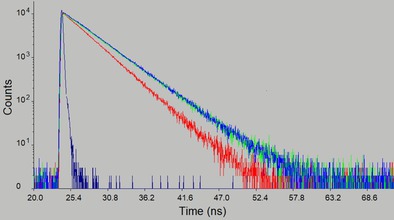

## 1 Introduction

Fluorescent dyes that are both small and bright in aqueous solutions are important in many fields of investigation and applications, where they are used as labels and stains to emit in the visible or infrared ranges of the spectrum. Examples of fields of application include optical microscopy, optical nanoscopy, in vivo imaging, nucleic acid detection and quantification, fluorescence in situ hybridization, cell sorters, flow cytometry, capillary electrophoresis, and high‐performance liquid chromatography, to mention a few. For a more complete overview, see Ref. [1].

The first excited state fluorescence mean‐lifetime (*τ*_f_) of small and bright fluorescent dyes ranges from about 0.1 to over 50 ns. This photophysical property is used in specific areas of applications, some of which are becoming of great importance. These include detection schemes based on single molecules, time‐resolved microscopy, time‐resolved spectroscopy, fluorescence depolarization, rotational relaxation measurements, two‐photon spectroscopy, and the real‐time imaging of fast‐moving targets [1].

In the present work, the fluorescence mean‐lifetimes of a series of small and bright fluorescent dyes were determined. Small in the sense that they are made of less than 50 non‐hydrogen atoms. This was done for many reasons. Firstly, because some of them were not yet reported in the literature. Secondly, because the *τ*_f_ of some dyes had only previously been measured in organic solvents or plain water, but not in aqueous buffered solutions. Finally, a few more dyes were added to the list as references for comparison with the literature.

## 2 Experimental

### 2.1 Reagents

The following reagents were purchased from Sigma (St. Louis, Missouri): 5‐aminofluorescein (cat. 201626), 5(6)‐carboxyfluorescein (FAM) (cat. 21877), 5(6)‐carboxy‐X‐rhodamine (ROX) (cat. 219654), 8‐aminopyrene‐1,3,6‐trisulfonic acid (cat. 09341), 8‐hydroxypyrene‐1,3,6‐trisulfonic acid (cat. H1529), acridine orange (cat. 318337), pyrene‐1,3,6,8‐tetrasulfonic acid (cat. 82658), Rho 6G 2^+^ (cat. 42514), Rho 6G 3^+^ (cat. 29297), acetic acid, sodium acetate, and potassium tetraborate. Additionally, the following were purchased from Fanbo (Beijing, China): 6‐HEX‐SE (cat. 202), 6‐TET‐SE (cat. 211), and TAMRA‐SE (cat. 376). Bodipy FL was purchased from Invitrogen (Eugene, Oregon).

### 2.2 Instrumentation

Fluorescence mean‐lifetimes were measured using a homemade experimental setup consisting of a regenerative amplified Yb:KGW femtosecond laser system with a 220 fs pulse width, operating at 343 nm (third harmonic of 1030 nm) and working at a 1‐kHz repetition rate. The laser beam was focused by a convergent lens, in which the samples were placed near the focal point. The fluorescence signal was collected perpendicularly to the excitation beam by an optical fiber that directed the signal to a photodetector with ~ 700 ps raise time. Between the optical fiber and the photodetector, an optical filter was used to avoid scattered excitation wavelength, only allowing fluorescence to be transmitted. To obtain the fluorescence lifetime, all studied samples were placed in a 2.0 mm optical path quartz cuvette at room temperature. The time‐resolved fluorescence signal of each sample was measured several times over 10 s at 100‐Hz repetition rate in a 1.0 GHz digital oscilloscope, following which the measurements were averaged. For each measured fluorescence lifetime, the instrument response function was also acquired. By convoluting this instrument response function with a single exponential function, the experimental fluorescence curve was adjusted and the fluorescence mean‐lifetime was calculated. The absorption spectra were measured using a Shimadzu model UV‐1800 spectrophotometer and fluorescence spectra were measured using a Hitachi spectrofluorometer model F‐7000.

### 2.3 Sample Preparation

**FIGURE 1:**
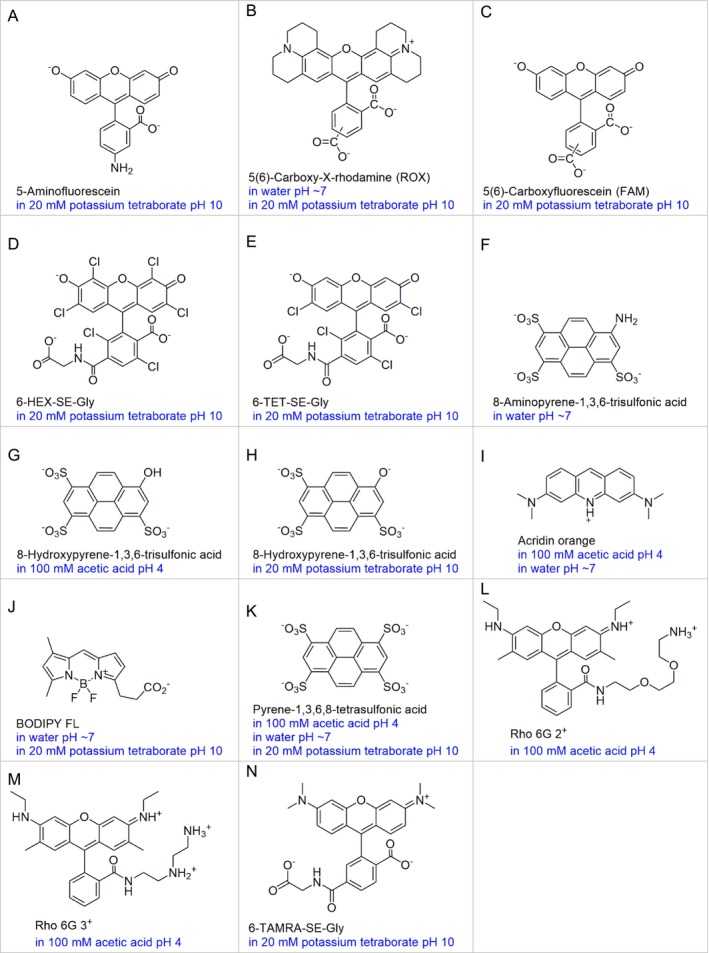
Molecular structures of the dyes. The names given correspond to their neutral forms whereas the structures shown correspond to their prevalent ionic form in the given solutions and pH (in blue). There are some controverses in the literature regarding the p*k*_a_ values of pyrene‐1,3,6,8‐tetrasulfonic acid. Therefore, we used the same ionic form in the three buffer solutions used (K).

The dyes were prepared at the concentration of 1 mM in the respective buffer solutions or water, as indicated in Figure 1. They were then further diluted with the respective solvents (buffer solutions or water) until the desired concentration for the measurements was reached (typically 10 to 100 μM). The molecular structure of the prevalent ionic form of the dyes at the indicated pH (blue legends) are also shown in Figure 1. Note that the names used throughout this work are the names of their neutral forms, while the measurements were taken when the dyes were in their ionic forms defined by the buffer solutions (see Figure 1).

The succinimidyl esters 6‐HEX‐SE, 6‐TET‐SE, and TAMRA‐SE were allowed to react with glycine (at a five‐fold concentration) at room temperature for 30 min prior the measurements (Figure 1D,E,N, respectively). However, no significant differences in the fluorescence mean‐lifetime are expected between these derivatives and the succinimidyl ester forms or even the carboxylic (or even carboxylate) forms of these dyes.

For clarity and unambiguity, the short names, IUPAC names and/or other names of the dyes are given in Table 1. The suppliers' product codes of the dyes used in the present work are also given.

**TABLE 1:** Short names, IUPAC names, and other names of the dyes studied in the present work. These names reflect the neutral forms of the molecules. It is of note that the measurements were taken while the molecules were in their ionic forms (see Figure 1).

Short name	IUPAC names or other names	Supplier catalog number
5‐Aminofluorescein	Fluoresceinamine isomer I	Sigma/Merck 201626
5 (6)‐ROX^1^	5 (6)‐Carboxy‐X‐rhodamine	Sigma/Merck 219654
5 (6)‐FAM^1^	5 (6)‐Carboxyfluorescein	Sigma/Merck 21877
6‐HEX‐SE‐Gly^1^	*N*‐Hydroxy‐succinimidyl ester of hexachlorocarboxyfluorescein (glycine derivative)	Fanbo 202
6‐TET‐SE‐Gly^1^	*N*‐Hydroxy‐succinimidyl ester of tetrachlorocarboxyfluorescein (glycine derivative)	Fanbo 211
8‐APTS	8‐Aminopyrene‐1,3,6‐trisulfonic acid	Sigma/Merck 09341
8‐HPTS	8‐Hydroxypyrene‐1,3,6‐trisulfonic acid	Sigma/Merck H1529
Acridine orange	*N*,*N*,*N*′,*N*′‐Tetramethylacridine‐3,6‐diamine or 3,6‐dimethylaminoacridine	Sigma/Merck 318337
BODIPY FL^1^	4,4‐Difluoro‐5,7‐dimethyl‐4‐bora‐3a,4a‐diaza‐s‐indacene‐3‐propanoic acid	Invitrogen
PTS	Pyrene‐1,3,6,8‐tetrasulfonic acid	Sigma/Merck 82658
Rho 6G 3^+^	*N*‐[2‐(2‐Aminoethylamino)ethyl] rhodime 6G‐amide bis (trifluoro acetate)	Sigma/Merck 29297
Rho 6G 2^+^	*N*‐(8‐Amino‐3,6‐dioxaoctyl)rhodamine 6G‐amide bis (trifluoroacetate)	Sigma/Merck 42514
TAMRA‐SE‐Gly^1^	*N*‐Hydroxy‐succinimidyl ester of carboxytetramethylrhodamine (glycine derivative)	Fanbo 376

^1^ BODIPY, FAM, TET, HEX, ROX, and TAMRA are trademarks of Thermo Fisher Scientific (Waltham, MA, USA).

## 3 Results

**FIGURE 2:**
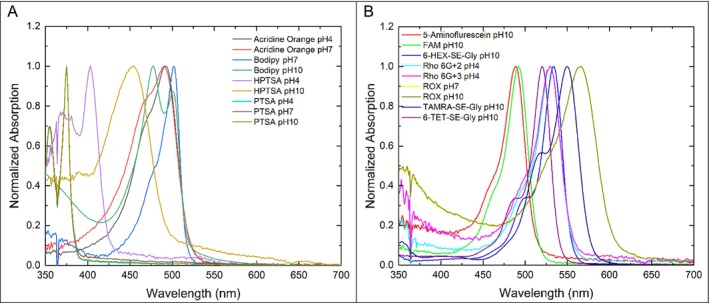
Normalized absorption spectra of the dyes in aqueous buffers.

**FIGURE 3:**
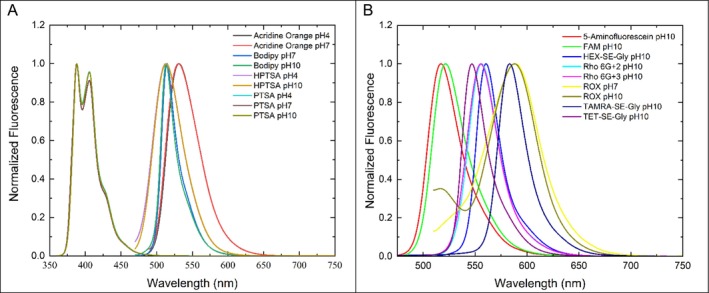
Normalized emission spectra of the dyes in aqueous buffers.

Figure 2 shows the normalized absorption spectra of the dyes in the aqueous buffered solutions used to measure the fluorescence mean lifetimes, and Figure 3 shows the corresponding normalized emission spectra. In the literature, these photophysical values are most often reported in plain water rather than in buffered aqueous solutions, which are the solutions most commonly used in capillary electrophoresis, HPLC, and cell sorters.

**FIGURE 4:**
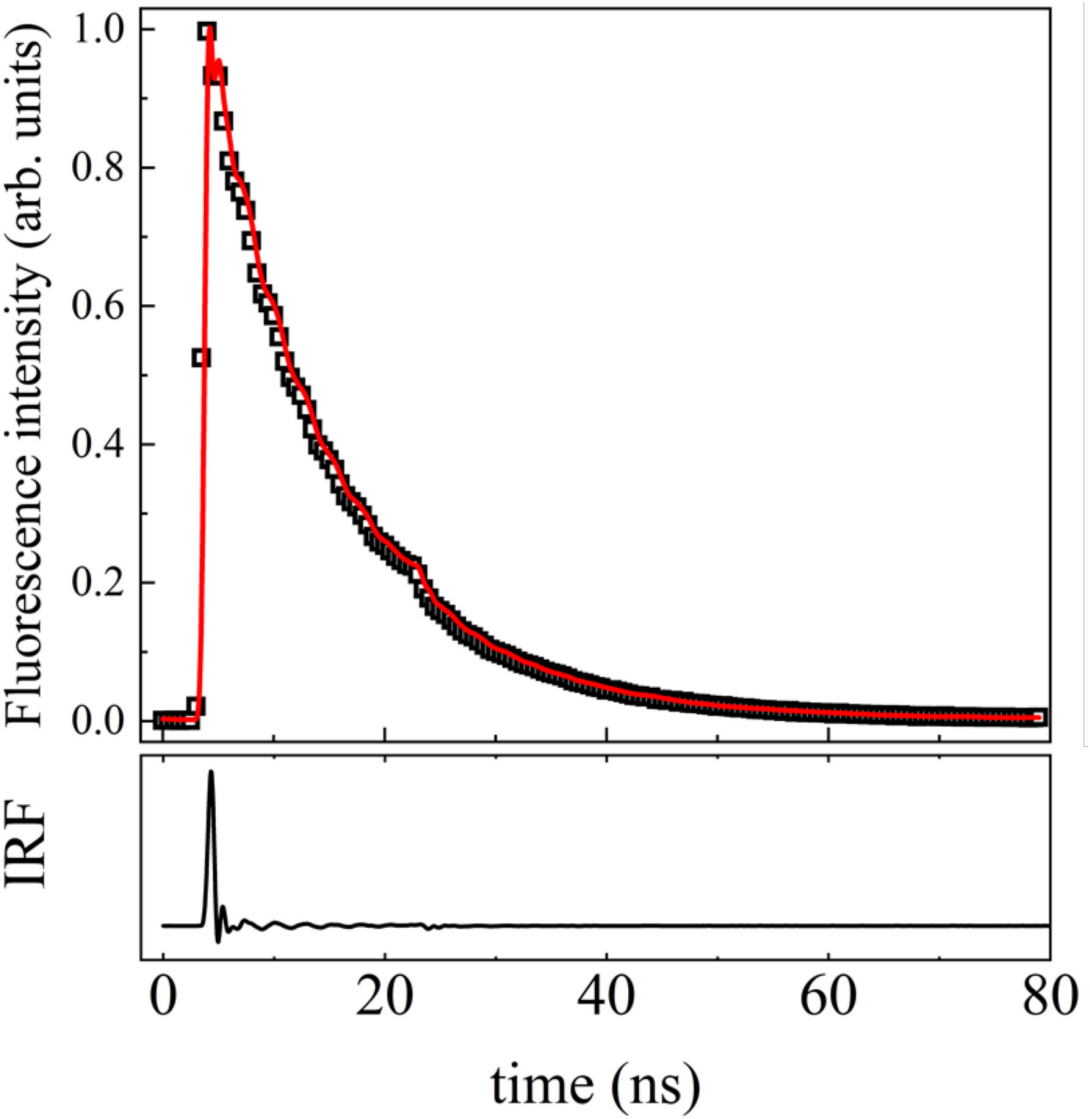
Time‐resolved fluorescence decay curve for pyrene‐1,3,6,8‐tetrasulfonate (open squares) and the respective fitting (red line) obtained by convoluting the instrument response function with a single exponential function. At the bottom, the solid black line represents the instrumental response function (IRF). The oscillation features are caused by the electronics of the technique.

Figure 4 shows one example of a typical measurement of fluorescence mean‐lifetime taken in the instrument of the present work, in which the experimental fluorescence decay curve (open squares) of pyrene‐1,3,6,8‐tetrasulfonate in neutral water can be seen, as well as the IRF (open circles) and the adjusted curve (solid red line). The measurement curves of the other dyes are shown in Fig. SI‐1 of the Supplementary Information text (SI).

The excitation and emission wavelengths used in the measurements using the regenerative amplified Yb:KGW femtosecond laser system and photocounter are shown in Table 2. These data are from Ref. [1] and the references therein. Note that when the longest wavelength of excitation maximum was not available, then the longest wavelength of absorption maximum was used. Both are simply referred as *λ*_x,max_ in Table 2.

**TABLE 2:** Photophysical properties of the dyes (taken from Ref. [1] and references therein). IUPAC names are given in Table 1. Where, *λ*_x,max_ is the longest wavelength of excitation maximum, *λ*_e,max_ is the emission maximum, Δ_S_ is the Stokes shift, *ε*_max_ is the molar absorption coefficient, Φ_f_ is the fluorescence quantum yield (a non‐dimensional quantity), and τ_f_ is the fluorescence mean‐lifetime.

Short names	Solvent	*λ*_x,max_	*λ*_e,max_	Δ_S_	*ε*_max_	Φ_f_	*B*_max_	τ_f_	Ref.
		nm	nm	nm	mM^−1^ cm^−1^	ndq	mM^−1^ cm^−1^	ns	
5‐Aminofluorescein	Water with 0.02 M 1,8‐Diaza‐biciclo[5,4,0] undec‐7‐eno	488	518	30	78.0	0.008	0.62	0.12 (98%), 3.89	[2, 3]
5 (6)‐Carboxy‐X‐rhodamine, 5 (6)‐ROX	Water pH < 7	576	601	25	82.0	0.70	57.4		[1, 4]
5 (6)‐Carboxyfluorescein, 5 (6)‐FAM	100 mM PBS pH 9	496	520	25	83.0	0.90	74.7		[1, 4]
6‐HEX	Water pH 9	535	559	24	98.0	0.70	68.6		[1, 4]
6‐TET	Water pH 9	521	536	15	73.0	0.70	51.1		[1, 4]
8‐Aminopyrene‐1,3,6‐trisulfonic acid (8‐APTS)	Water	424	505	81	20.6	0.90	18.5		[5, 6]
8‐Hydroxypyrene‐1,3,6‐trisulfonic acid (8‐HPTS)	Water pH 2	403	443	40	20.0	0.95	19.0	4.8	[5, 7, 8]
8‐Hydroxypyrene‐1,3,6‐trisulfonic acid (8‐HPTS)	Water pH 10	460	514	54	24.0	0.95	22.8	5.3	[5, 7, 8]
Acridine orange	Water	488	528	40	50.0	0.12	6.0		[9, 10]
Acridine orange	HEPES pH 7.3	493	528	35	50.0	0.21	10.5		[9, 10]
BODIPY FL	Water	502	511	9	92.0	0.97	89.2		[1, 4]
Pyrene‐1,3,6,8‐tetrasulfonic acid (PTS)	Water pH 7	374	403	29	51.0	0.56	28.5	12.5	[1, 5, 8]
Rho 6G 2^+^	Water pH < 7	530	553	23	101.0	0.93	93.9		[1]
Rho 6G 3^+^	Water pH < 7	529	551	22	101.0	0.91	91.9		[1]
TAMRA	Water pH 7	546	576	30	90.0	0.20	18.0		[4, 7]
TAMRA	50 mм PBS pH 8–9	547	574	27	77.0	0.35	26.9	2.2	[4, 7]

The results of the measurements at room temperature using the regenerative amplified Yb:KGW femtosecond laser system and photocounter detection are shown in Table 3. Note that plain water is the most used aqueous solutions in the literature (Table 2), which differs from the pH 4, 7, and 10 buffer solutions used in the present work (Table 3). These latter are more compatible with capillary electrophoresis, HPLC, and cell sorters.

**TABLE 3:** Fluorescence mean‐lifetimes (τf) and respective standard deviations (SD) of dyes measured in the present work. The molecular structures of the prevalent ionic forms of these dyes are shown in Figure 1. Other photophysical parameters of the dyes are given in Table 2.

#	Short name or acronym	Buffer solution or solvent used	*τ*_f_ (ns)	SD (ns)	Φ_f_
1	5‐Aminofluorescein	20 mM potassium tetraborate pH 10	3.4	0.1	0.24
2	5 (6)‐Carboxy‐X‐rhodamine, 5 (6)‐ROX	20 mM potassium tetraborate pH 10	4.3	0.2	0.70
3	5 (6)‐Carboxy‐X‐rhodamine, 5 (6)‐ROX	Water pH ~ 7	3.0	0.3	0.70
4	5 (6)‐Carboxyfluorescein, 5 (6)‐FAM	20 mM potassium tetraborate pH 10	7.4	0.3	0.90
5	6‐HEX‐SE‐Gly	20 mM potassium tetraborate pH 10	6.5	0.2	0.61
6	6‐TET‐SE‐Gly	20 mM potassium tetraborate pH 10	8.2	0.3	
7	8‐Aminopyrene‐1,3,6‐trisulfonic acid, 8‐APTS	Water pH ~ 7	5.6	0.1	
8	8‐Hydroxypyrene‐1,3,6‐trisulfonic acid, 8‐HPTS	20 mM potassium tetraborate pH 10	5.9	0.3	
9	8‐Hydroxypyrene‐1,3,6‐trisulfonic acid, 8‐HPTS	100 mM acetic acid/sodium acetate pH 4	5.0	0.2	
10	Acridine orange	100 mM acetic acid/sodium acetate pH 4	8.9	0.3	
11	Acridine orange	Water pH ~ 7	6.8	0.5	
12	BODIPY FL	20 mM potassium tetraborate pH 10	6.6	0.2	
13	BODIPY FL	Water pH ~ 7	10.3	0.3	
14	Pyrene‐1,3,6,8‐tetrasulfonic acid, PTS	20 mM potassium tetraborate pH 10	12.4	0.7	
15	Pyrene‐1,3,6,8‐tetrasulfonic acid, PTS	100 mM acetic acid/sodium acetate pH 4	13.0	0.6	
16	Pyrene‐1,3,6,8‐tetrasulfonic acid, PTS	Water pH ~ 7	12.4	0.6	
17	Rho 6G 2^+^	100 mM acetic acid/sodium acetate pH 4	5.2	0.1	0.91
18	Rho 6G 3^+^	100 mM acetic acid/sodium acetate pH 4	5.4	0.2	0.93
19	TAMRA‐SE‐Gly	20 mM potassium tetraborate pH 10	1.6	0.3	0.24

## 4 Discussion

5‐Aminofluorescein is a fluorescein derivative used to label reducing carbohydrates [2]. Its photophysical properties change significantly upon derivatization; nevertheless, its *τ*_f_ (SD) was measured in 20 mM potassium tetraborate pH 10. The ionic form of this dye in this buffer solution is shown in Figure 1A. Here, it was found to have *τ*_f_ = 3.4 ns with SD = 0.1 ns, which is similar to values found in the literature [3].

5(6)‐Carboxy‐X‐rhodamine (ROX) is a well‐known rhodamine derivative [4] that is widely used as a label in molecular biology and in both DNA sizing and sequencing. In water pH 7 and 20 mM potassium tetraborate pH 10, this dye exhibits the ionic form shown in Figure 1B. In these solutions, its *τ*_f_ (SD) is 4.4 ns (0.2 ns) and 3.0 (0.3 ns), respectively.

5(6)‐Carboxyfluorescein (FAM) is a fluorescein derivative that is also widely used as a label in molecular biology and in both DNA sizing and sequencing [4]. In 20 mM potassium tetraborate (ionic form shown in Figure 1C), it was found to have *τ*_f_ = 7.4 ns with SD = 0.3 ns.

6‐HEX and 6‐TET also belong to the class of fluorinated fluorescein dyes. The succinimidyl moiety (6‐HEX‐SE and 6‐TET‐SE) is used to label these dyes to primary amine and secondary amine containing targets. As in the previous cases, these dyes are used as labels in molecular biology and in both DNA sizing and sequencing [4]. In the present work, they were labeled to glycine prior to measurement (the structures of 6‐HEX‐SE‐Gly and 6‐TET‐SE‐Gly are shown in Figure 1D,E, respectively). The photophysical properties of these dyes (Table 2) are not expected to change upon labelling to most small dyes that do not absorb in the visible range of the spectrum.

8‐Aminopyrene‐1,3,6‐trisulfonic acid (8‐APTS) is used to label reducing carbohydrates, similar to 5‐aminofluorescein. However, 8‐APTS shows superior photostability and higher electrophoretic mobility in aqueous solutions, which gives higher separation efficiencies [5, 6]. In water pH ~ 7 (Figure 1F), *τ*_f_ = 5.6 ns was found with SD = 0.1 ns for this dye.

The fluorescence mean‐lifetime of 8‐hydroxypyrene‐1,3,6‐trisulfonic acid was measured in 100 mM acetic acid/sodium acetate pH 4 (Figure 1G) and in 20 mM potassium tetraborate pH 10 (Figure 1H). It was not measured at neutral pH because the dye is a mixture of two ionic forms in this pH range (the p*k*_a_ of the phenol group is 7.3 [1]). The values of τ_f_ (SD) found are 5.0 ns (0.2 ns) and 5.9 ns (0.3 ns), in pH 4 and 10, respectively. These values are close to the values found in the literature (where pure water is used instead of a buffer solution) [5, 7, 8]. Note that the other photophysical properties of these two ionic forms (in acidic and basic pH) are significantly different (summarized in Table 2).

Acridine orange (3,6‐dimethylaminoacridine) belongs to the acridine class. The p*K*_a_ of acridine is 9.8 [9, 10] (Figure 1I); therefore, *τ*_f_ was measured in water pH ~ 7 and 100 mM acetic acid/sodium acetate pH 4. The values of τ_f_ (SD) found are 8.9 ns (0.3 ns) and 6.8 ns (0.5 ns), respectively.

BODIPY FL belongs to the class of boron‐dipyrromethene, also called 4,4‐difluoro‐4‐bora‐3a,4a‐diaza‐s‐indacene. Dyes in this class are small, bright, and exhibit a small Stokes shift [4]. The stokes shift of BODIPY FL (Figure 1J) was confirmed to be only 9 nm [1]. The *τ*_f_ (SD) found in the present measurements were 6.6 ns (0.2 ns) in 20 mM potassium tetraborate and 10.3 ns (0.3) in water pH ~ 7.

Pyrene‐1,3,6,8‐tetrasulfonic acid has four p*K*_a_ values. The values are expected to be low and, in addition to this, a negligible difference in *τ*_f_ is expected when it goes from ionized to neutral, as the resulting ─O^−^ is two covalent bonds apart from the fluorescent structure (pyrene ring) [1, 5, 8]. Therefore, *τ*_f_ is almost the same at pH 4, ~ 7, and 10, as shown in Table 3 (entries 15, 16, and 17). The values of *τ*_f_ (SD) measured are 12.4 ns (0.7 ns), 13.0 ns (0.6 ns), and 12.4 ns (0.7 ns), respectively.

Rho 6G 2^+^ is a rhodamine derivative that is only soluble in aqueous solution with pH 5 or lower. Therefore, *τ*_f_ (SD) was measured in 100 mM acetic acid/sodium acetate pH 4 (Figure 1L). The *τ*_f_ (SD) found is 5.2 ns (0.1 ns) in 100 mM acetic acid/sodium acetate pH 4.

Rho 6G 3^+^ is also a rhodamine derivative that is only soluble in acidic pH. Therefore, *τ*_f_ (SD) was also measured in 100 mM acetic acid/sodium acetate pH 4 (Figure 1M). This dye is rare as it is small, bright, highly photostable, and highly positively charged (tricationic) in acidic aqueous solutions. The value of *τ*_f_ (SD) found is 5.4 ns (0.2 ns). As for Rho 6G 2^+^, there is no data in the literature for this dye other than in Ref. [1].

TAMRA is a bright rhodamine. Its succinimidyl ester variant (TAMRA‐SE) may exhibit small photophysical differences compared to its bare dye [4, 7]. In the present work, the measurement of τ_f_ (SD) was taken in 10 mM potassium tetraborate pH 10 using its glycine derivative (TAMRA‐SE‐Gly, see Figure 1N). Its τ_f_ (SD) value was found to be 1.6 ns (0.3 ns).

The present data showed a 5‐aminofluorescein much brighter in 20 mM potassium tetraborate pH 10 (Φ_f_ = 0.24) compared to water with 0.02 M 1,8‐Diaza‐biciclo[5,4,0] undec‐7‐eno (0.008) [2, 3]. The brightness of FAM, ROX, and HEX is similar to what is found in the literature [1, 4]. The Φ_f_ of Rho 6G 2^+^ and Rho 6G 3^+^ is identical to what is found in the literature [1].

## 5 Conclusion

The fluorescence mean‐lifetime of a series of small and bright fluorescent dyes were determined in one or more aqueous solution and published for the first time in the present work. The measured fluorescence mean‐lifetime of some dyes is in good agreement with what is found in the literature despite differences in the buffer solutions used and, eventually, on the temperature of the measurements.

The rhodamines (TAMRA, ROX, Rho 6G 2^+^, and Rho 6G 3^+^) exhibited the smallest values of fluorescence mean‐lifetime, followed by the fluorescein derivatives and BODIPY derivatives. The longest fluorescence mean‐lifetimes were observed among pyrene‐1,3,6,8‐tetrasulfonic acids, with *τ*_f_ larger than 12 ns.

## Author Contributions

**Leonardo De Boni:** methodology, software, investigation. **Klester dos Santos Souza:** investigation. **Melissa Machado Rodrigues:** investigation. **Bruna Nitzke Minuzzi:** investigation. **Marcelo Barbalho Pereira:** investigation, methodology. **Milton Katsumi Sasaki:** investigation, methodology. **Diogo Seibert Ludtke:** conceptualization. **Tarso B. Ledur Kist:** conceptualization, writing – original draft, supervision, writing – review and editing.

## Funding

There was no specific funding for this work.

## Conflicts of Interest

The authors declare no conflicts of interest.

## Supporting information


**Figure S1:** Fluorescence decay curves (open circles) of all samples studied. The solid black lines represent the instrument response function (IRF) and the solid red lines are the fitting obtained by using the convolution method.

## Data Availability

The data that support the findings of this study is available from the author upon reasonable request.
