# Supplementary material for: Fluorescence Mean‐Lifetimes of a Series of Small and Bright Fluorescent Dyes
Source: Luminescence. 2026 Jan 8;41(1):e70392. doi: 10.1002/bio.70392 (PMC12783449; doi:10.1002/bio.70392)
Supplement: Supplementary file 1 — Figure S1: Fluorescence decay curves (open circles) of all samples studied. The solid black lines represent the instrument response function (IRF) and the solid red lines are the fitting obtained by using the convolution method. [file BIO-41-e70392-s001.pdf]

Luminescence

WILEY

LUMINESCENCE
The Journal of Biological and Chemical Luminescence

SUPPLEMENTARY INFORMATION

Fluorescence Mean-lifetimes of a Series of Small and Bright Fluorescent Dyes

Leonardo De Boni[1] | Klester dos Santos Souza[2] | Melissa Machado Rodrigues[3] | Bruna Nitzke Minuzzi[4] | Marcelo Barbalho Pereira[5] | Milton Katsumi Sasaki [2] | Diogo Seibert Lüdtke[2] | Tarso B. Ledur Kist[2,6*].

[1] Instituto de Física de São Carlos, Universidade de São Paulo, São Carlos, SP, Brasil.
[2] Instituto de Química, Universidade Federal do Rio Grande do Sul, Porto Alegre, RS, Brasil.
[3] Ciências Exatas e Engenharias, Universidade de Caxias do Sul, Caxias do Sul, RS, Brasil.
[4] Instituto de Ciências Básicas da Saúde, Departamento de Bioquímica, Universidade Federal do Rio Grande do Sul, Porto Alegre, RS, Brasil
[5] Instituto de Física, Universidade Federal do Rio Grande do Sul, Porto Alegre, RS, Brasil.
[6] LSO/PEA, Escola Politécnica, Universidade de São Paulo, São Paulo, SP, Brasil.

Table of Content

1. Introduction 1

2. Fluorescence decay curves of all samples 2

1 | Introduction

The fluorescence lifetimes of the samples were measured using a custom-built time-resolved fluorescence setup. This system employs the third harmonic (343 nm) of a femtosecond amplified laser operating at 1030 nm, delivering pulses of approximately 220 fs. The repetition frequency is adjustable from a few Hertz up to the kHz range. A 30 cm focal length converging lens focuses the beam, producing an intensity gradient that allows the sample to be placed at the appropriate irradiation level. The resulting fluorescence is captured through an optical fiber coupled to a high-speed silicon photodetector with a temporal resolution of roughly 0.7 ns. The time-dependent fluorescence signal is monitored in real time using a gigahertz-bandwidth digital oscilloscope. To extract the fluorescence lifetime from the experimental data, our in-house software applies a mathematical convolution between the recorded signal and the instrument response function (IRF). This follows the relation $I_{measured} = I_{real} * \text{IRF}$, where $I_{measured}$ represents the detected fluorescence and I_{real} corresponds to the true fluorescence decay. In the final step, the actual fluorescence decay (τ_F) is determined from I_{real}. For every measurement, the IRF is obtained separately by detecting only the laser's scattered light, ensuring no contribution from sample fluorescence. The experimental decay traces ($I_{measured}$), the corresponding IRF signals, and the resulting convoluted curves (I_{real}) for all samples are presented in Fig. SI-1. It should be noted that each sample was measured 10 times, with every individual measurement representing an average of roughly 1000 laser pulses acquired at a 100 Hz repetition rate, totaling about 10 seconds per acquisition. The method is validated using reference compounds with well-established lifetimes, including Rhodamine 6G, Rhodamine B, Fluorescein, and Tetraphenyl Porphyrin.

2 | Fluorescence decay curves of all samples

The decay curves of all samp-les studied in the present work are given in Fig. SI-1.

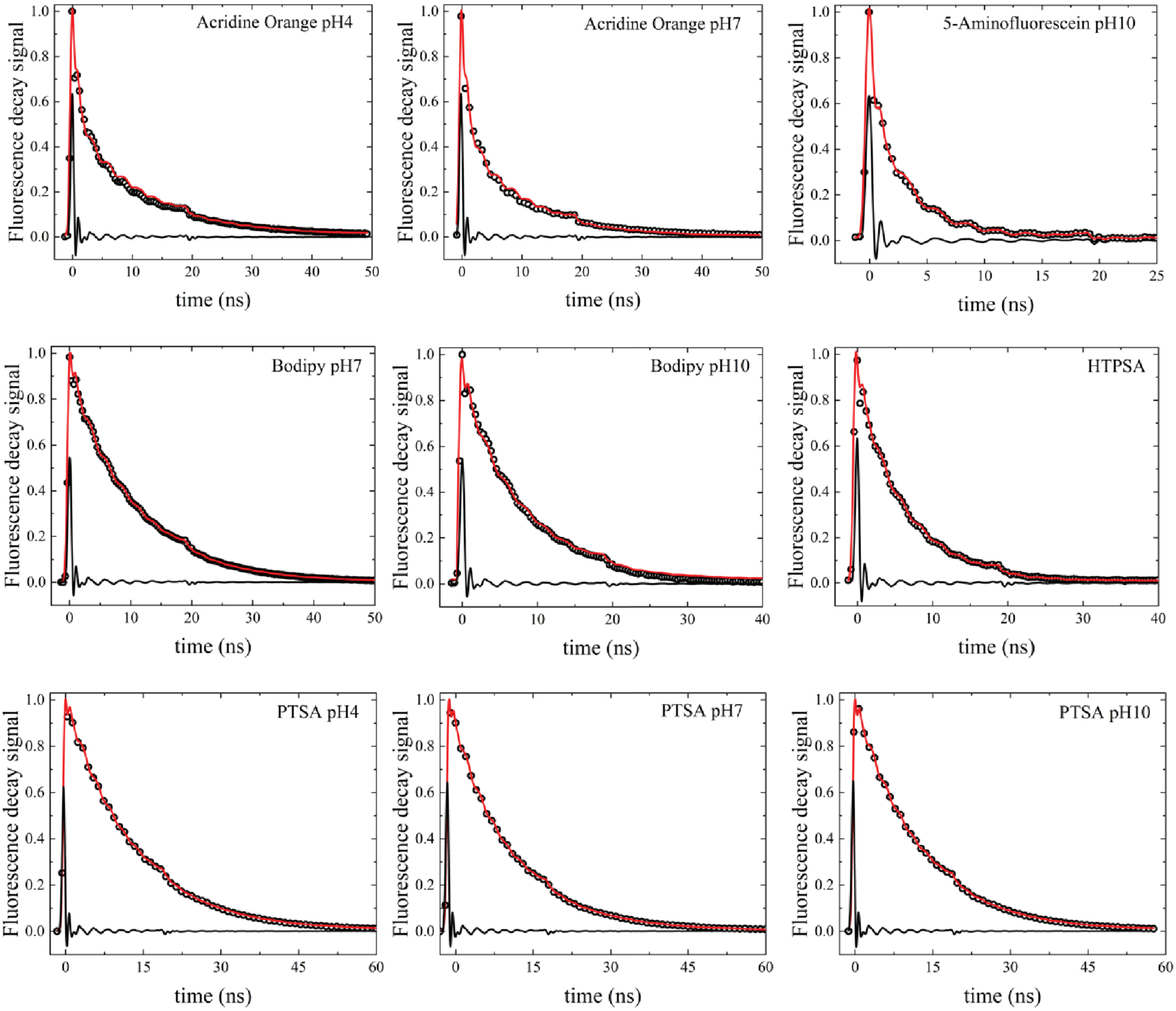

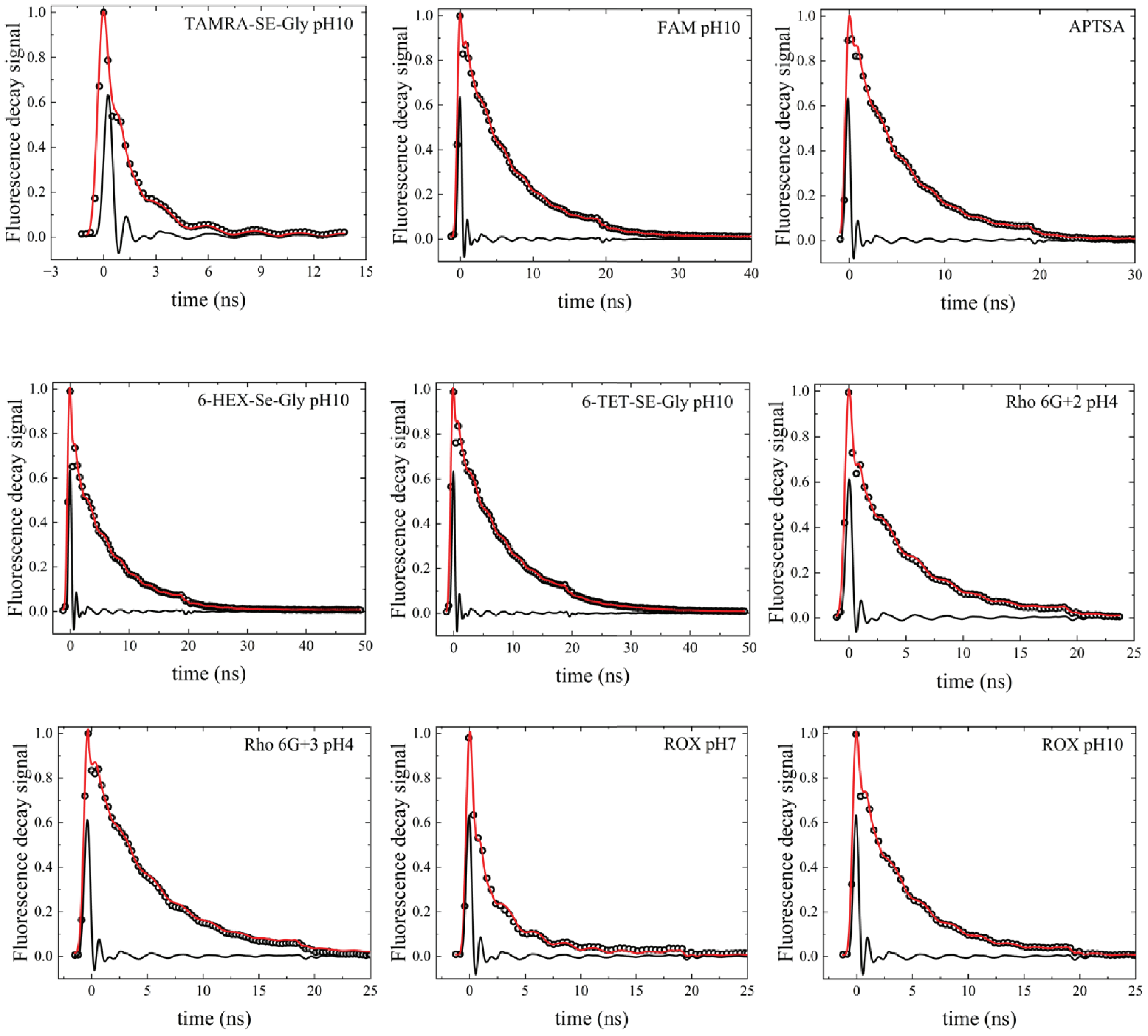

Figure SI-1. Fluorescence decay curves (open circles) of all samples studied. The solid black lines represent the instrument response function (IRF) and the solid red lines are the fitting obtained by using the convolution method.
